# Thrombolysis in acute ischemic stroke in patients with dementia: A Swedish registry study

**DOI:** 10.1212/WNL.0000000000004598

**Published:** 2017-10-31

**Authors:** Eva Zupanic, Mia von Euler, Ingemar Kåreholt, Beatriz Contreras Escamez, Johan Fastbom, Bo Norrving, Dorota Religa, Milica G. Kramberger, Bengt Winblad, Kristina Johnell, Maria Eriksdotter, Sara Garcia-Ptacek

**Affiliations:** From the Karolinska Institutet (E.Z., D.R., B.W.), Department of Neurobiology, Care Sciences and Society, Center for Alzheimer Research, Division of Neurogeriatrics, Huddinge, Sweden; Department of Neurology (E.Z., M.G.K.), University Medical Centre, Ljubljana, Slovenia; Karolinska Institutet (M.v.E.), Department of Clinical Science and Education, Södersjukhuset, and Department of Medicine, Solna; Karolinska University Hospital (M.v.E.), Department of Clinical Pharmacology; Karolinska Institutet and Stockholm University (I.K., J.F., K.J.), Aging Research Center, Stockholm, Sweden; Jönköping University (I.K.), Institute of Gerontology, School of Health and Welfare, Aging Research Network–Jönköping; Karolinska Institutet (B.C.E., M.E., S.G.-P.), Department of Neurobiology, Care Sciences and Society, Center for Alzheimer Research, Division of Clinical Geriatrics, Huddinge, Sweden; Department of Geriatrics (B.C.E.), Hospital Universitario de Getafe, Madrid, Spain; Lund University (B.N.), Skane University Hospital, Department of Clinical Sciences Lund, Neurology; Karolinska University Hospital, Department of Geriatric Medicine (D.R., M.E., S.G.-P.); and Södersjukhuset (S.G.-P.), Department of Internal Medicine, Section for Neurology, Stockholm, Sweden.

## Abstract

**Objective:**

To compare access to intravenous thrombolysis (IVT) for acute ischemic stroke (AIS) and its outcomes in patients with and without dementia.

**Methods:**

This was a longitudinal cohort study of the Swedish dementia and stroke registries. Patients with preexisting dementia who had AIS from 2010 to 2014 (n = 1,356) were compared with matched patients without dementia (n = 6,755). We examined access to thrombolysis and its outcomes at 3 months (death, residency, and modified Rankin Scale [mRS] score). Odds ratios (ORs) and 95% confidence intervals (CIs) were calculated with logistic and ordinal logistic regression.

**Results:**

The median age at stroke onset was 83 years in both groups. IVT was administered to 94 (7.0%) patients with dementia and 639 (9.5%) patients without dementia. The OR of receiving IVT was 0.68 (95% CI 0.54–0.86) for patients with dementia. When the analysis was repeated exclusively among patients independent in everyday activities, dementia status was no longer significant (OR 0.79, 95% CI 0.60–1.06). However, differences persisted in patients ≤80 years of age (OR 0.58, 95% CI 0.36–0.94). In patients who received thrombolysis, the incidence of symptomatic intracerebral hemorrhage (sICH; 7.4% vs 7.3%) and death at 3 months (22.0% vs 18.8%) did not differ significantly between the 2 groups. However, mRS score and accommodation status were worse among patients with dementia after 3 months in adjusted analyses (both *p* < 0.001). Unfavorable outcomes with an mRS score of 5 to 6 were doubled in patients with dementia (56.1% vs 28.1%).

**Conclusions:**

Younger patients with dementia and AIS are less likely to receive IVT. Among patients receiving thrombolysis, there are no differences in sICH or death, although patients with dementia have worse accommodation and functional outcomes at 3 months.

Dementia is not a contraindication for intravenous thrombolysis (IVT) in acute ischemic stroke (AIS), but most of the thrombolysis studies to date excluded or underrepresented octogenarians and nonagenarians,^1,2^ and guidelines differ in their recommendations for IVT use in this population. According to the American Heart Association/American Stroke Association, patients with dementia may benefit from IVT; however, decisions should be made individually with premorbid functional level taken into account.^3^ Alteplase is not indicated in patients >80 years of age according to the European Medicines Agency,^4^ while it is recommended in selected patients according to European stroke guidelines.^5^ Because of this inconsistency and fear of cerebral hemorrhage, physicians may adopt a more conservative approach in patients with dementia.^2,6^ Indeed, patients with dementia had increased mortality and poorer functional outcomes after stroke regardless of the use of reperfusion therapy in some^7,8^ but not other studies.^9,10^ Patients with dementia are less likely to receive IVT,^8,10^ even though increased incidence of symptomatic intracranial hemorrhage (sICH) has so far not been reported.^7,9,10^

There is a lack of recent data on use and outcomes of IVT in patients with dementia, and this subject has been identified as a high-priority research area.^3^ Our aim is to analyze the use and outcomes of IVT for AIS in patients with preexisting dementia in a large national cohort to determine whether dementia status is associated with lower use or poorer outcomes after IVT.

## METHODS

### Quality registries and study population.

We performed a longitudinal cohort study of patients diagnosed with dementia who subsequently had a first AIS. Patients with dementia were identified from Swedish Dementia Registry (SveDem), which has previously been described.^11,12^ Registration occurs at the time of dementia diagnosis with information on dementia type, demographics, and living situation.

Occurrence of AIS was identified with Riksstroke, the Swedish national quality registry for acute vascular diseases of the brain, one of the world's largest stroke registries.^13^ All hospitals admitting acute stroke patients participate, and coverage for ischemic stroke is >90%.^14^ Information on the registries is available at svedem.se and riksstroke.org.

Data on medication therapy from 2005 were obtained from the Swedish Prescribed Drug Register, including all prescription medications dispensed at Swedish pharmacies, with coverage of ≈100%.^15^ Comorbidities were collected from the Swedish National Inpatient Register, available from 1998 and coded according to the ICD-10, at present covering all in-hospital and specialist clinic diagnoses.^16^

Patients with a dementia diagnosis who subsequently had AIS were selected and matched by age (±3 years), sex, year of stroke, and geographic region with controls without dementia from Riksstroke. Controls without dementia were excluded if they ever had a SveDem registration, ever were diagnosed with dementia or confusional syndrome (ICD-10 code F00-F09 or G30-G32), or ever had taken antidementia medication (Anatomical Therapeutic Chemical [ATC] codes N06DX and N06DA, including donepezil, rivastigmine, galantamine, and memantine).

Of 58,154 patients registered in SveDem between May 2007 and December 2014, a total of 2,233 patients with dementia subsequently had AIS and were matched to 8,963 dementia-free controls from Riksstroke. The patient selection process is illustrated in the figure. Because of the changes in the IVT treatment window to 4.5 hours in 2009, data from 2010 to 2014 were used, and a study population of 1,356 AIS patients with dementia and 6,755 AIS controls without dementia was available for analyses.

**Figure:**
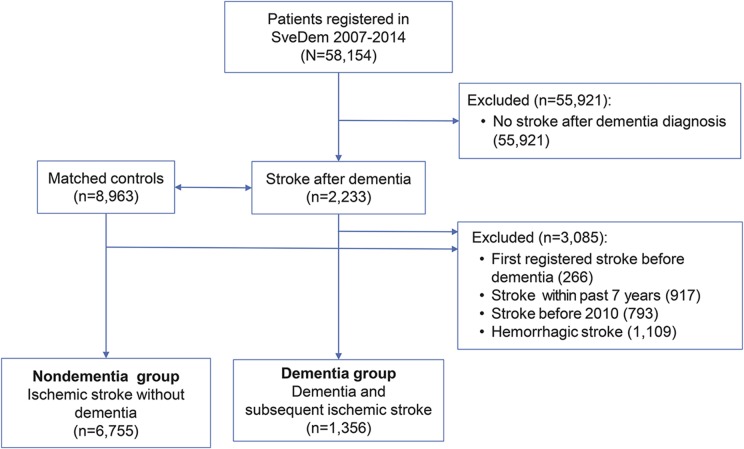
Patient selection process We excluded patients with AIS registered in Riksstroke before dementia, patients who had a history of stroke in the last 7 years according to the National Inpatient Register, patients with stroke before the year 2010 because of incomplete outcome variables and changes in the IVT treatment window to 4.5 hours in 2009, and patients with hemorrhagic stroke. AIS = acute ischemic stroke; IVT = intravenous thrombolysis; Riksstroke = Swedish national quality registry for acute vascular diseases of the brain; SveDem = Swedish Dementia Registry.

### Variables.

From SveDem, we used information on dementia type, date, and cognitive evaluation with the Mini-Mental State Examination (MMSE).

From Riksstroke, we used data on stroke event, demographics, living arrangements, transport to the hospital, activation of stroke code (protocol for management of acute stroke), IVT treatment, symptom-to-needle time (time interval between the symptom onset and initiation of the IVT), follow-up, and death. Independence in activities of daily living (ADL) before and after stroke was defined as independence in mobility, dressing, and toileting. Urban and rural typology was defined for Swedish county councils (“landsting”) on the basis of Organisation for Economic Co-operation and Development methodology.^17^ We used 2 clinical assessment tools as proxies for stroke severity: level of consciousness at admission to the hospital, determined with Reaction Level Scale (RLS), in which patients with RLS score of 1 are defined as alert, those with RLS score of 2 to 3 are defined as lethargic, and those with RLS score of 4 to 8 are defined as unconscious, and the NIH Stroke Scale (NIHSS). sICH is defined in Riksstroke as clinical worsening with an increase of ≥4 NIHSS points in the presence of intracranial hemorrhage starting <36 hours after the start of thrombolysis (nonsymptomatic intracranial hemorrhage is not recorded). Modified Rankin Scale (mRS) is a scale measuring degree of disability. Because mRS is a standard assessment method in stroke research and is missing in the Riksstroke registry, mRS score was estimated by translation of 5 self-reported Riksstroke functional outcome variables. This previously described conversion method offers high precision but cannot differentiate between functional groups with an mRS score of 0 to 2.^18^ Conversion was possible for mRS only after (and not before) stroke.

Number of medications was defined as the sum of all prescription drugs dispensed from pharmacies during the 3 months preceding the stroke, obtained from the Swedish Prescribed Drug Register, and was used as a proxy for comorbidity.^12^

### Statistical analysis.

For categorical variables, data are presented as number of cases and percentages. Continuous variables were summarized as mean ± SD or median (interquartile range). For calculating significant differences, the Student *t* test and Mann-Whitney *U* test were used for continuous and χ^2^ or Fisher exact tests for categorical variables.

Multivariate logistic regression analyses were used to assess the relationship between dementia status and receiving IVT. We assessed different outcomes after IVT: using multivariate logistic regression for (1) death at 3 months, (2) nursing home placement at 3 months, and (3) mRS score of 4 or 5 (vs mRS score <4) at 3 months and using multivariate ordinal logistic regression for (4) mRS score at 3 months as an ordinal scale of step-wise increase in mRS score. For mRS score, we tested the proportional odds/parallel-lines assumption using the STATA command GOLOGIT2 with a gamma parameterization. No significant violations of the assumptions were found.

Initial regression models were adjusted for age and sex. Next, we tested contraindications to IVT, possible confounding living arrangements, comorbidities, and medication, adding covariates in a stepwise manner. Covariates were chosen to reflect a burden of comorbidity, which might affect the decision to thrombolyse or, in the case of living situation, could affect time to hospital arrival and obtaining information. Individual drugs were subtracted from total number of medications if also used as separate confounders. We tested several interactions between comorbidities and medication. We tested all variables with *p* ≤ 0.25 significance in the univariate models. Variables that did not reach the significance of *p* < 0.05 in a multivariate model, did not change the β coefficient by ≈20%, or did not improve the *R*^2^ of the model were discarded. Adjusted odds ratios (ORs) with 95% confidence intervals (CIs) are presented. Post hoc analyses including NIHSS are also shown.

Post hoc propensity score–adjusted models were conducted. The propensity scores were obtained from multiple logistic regression for dementia status including the variables age, sex, smoking, number of medication, antiaggregants, antipsychotics, antidepressants, lipid-lowering agents, antihypertensives, atrial fibrillation, diabetes mellitus, hip fracture, ischemic heart disease, heart failure, renal failure, and liver failure (variables as shown in table 1).

**Table 1:**
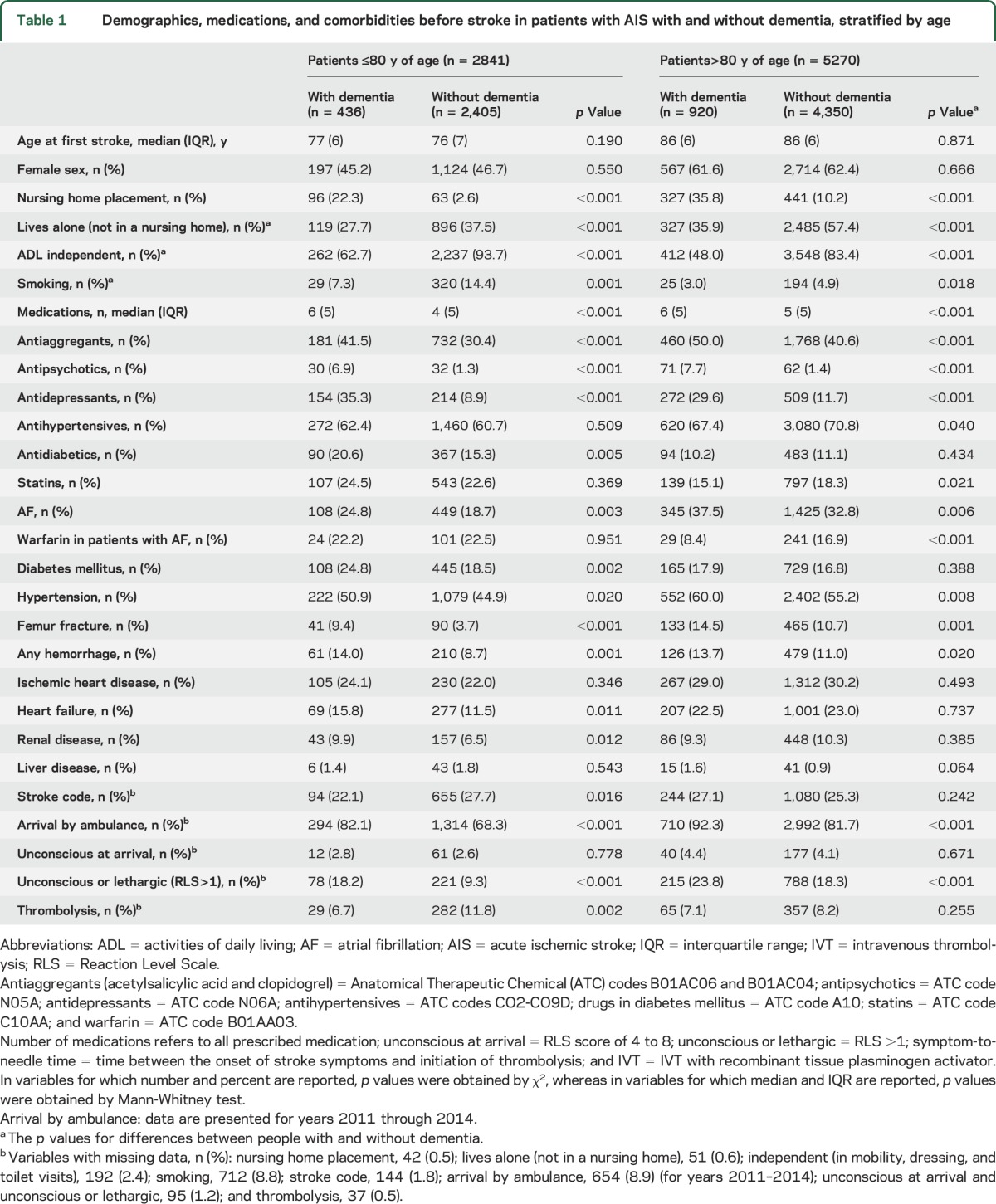
Demographics, medications, and comorbidities before stroke in patients with AIS with and without dementia, stratified by age

Tests were 2 tailed with a value of *p* < 0.05 considered significant. The IBM Statistical Package for Social Sciences for Windows, Sciences Software version 23 (IBM Corp, Armonk, NY) and STATA version 14 (StataCorp, College Station, TX) were used.

### Standard protocol approvals, registrations, and patient consents.

This study complies with the Declaration of Helsinki and was approved by the regional ethics review board in Stockholm, Sweden (dnr 2015/743-31/4). Patients and relatives were informed of inclusion in the registries at the time of diagnosis and could decline participation or withdraw consent. Data were deidentified before analysis.

## RESULTS

### Characteristics of the study population.

Of 8,111 patients with AIS, 1,356 patients had preexisting dementia and 6,755 patients did not have dementia registered. Because national Swedish stroke guidelines did not recommend IVT in patients >80 years of age until 2014 (except in particular cases after careful consideration),^19^ we age-stratified the cohort (table 1).

In the dementia group, at the time of the registration in SveDem, median MMSE score was 21 (5), which is in line with previous SveDem studies.^12,20^ Median time from dementia diagnosis to stroke was 546 days (705 days), and total days at risk were 894,439. Alzheimer dementia and mixed dementia were the most common dementia types (n = 628, 46.3%), and 311 patients (22.9%) had vascular dementia (results not presented).

### Use of thrombolysis.

Of all 8,111 patients, 733 (9.0%) received IVT, 94 (7.0%) patients with dementia and 639 (9.5%) patients without dementia. Patients with dementia were less often independent before stroke (52.8% vs 87.1%, *p* < 0.001). Patients who received IVT were more often independent than their counterparts who did not receive IVT (90.5% ADL independence in IVT group vs 80.7% in non-IVT group, *p* < 0.001; results not presented).

Patients with dementia treated with IVT were older (median age 83 vs 81 years, *p* = 0.016) and received more medications (median 6 vs 4, *p* < 0.001) than IVT-treated controls. Apart from ischemic heart disease, there were no other important differences in comorbidities. Other characteristics are shown in table 2. There were no differences between the 2 thrombolysis groups in the geographic county of origin or urban and rural groups (results not presented).

**Table 2:**
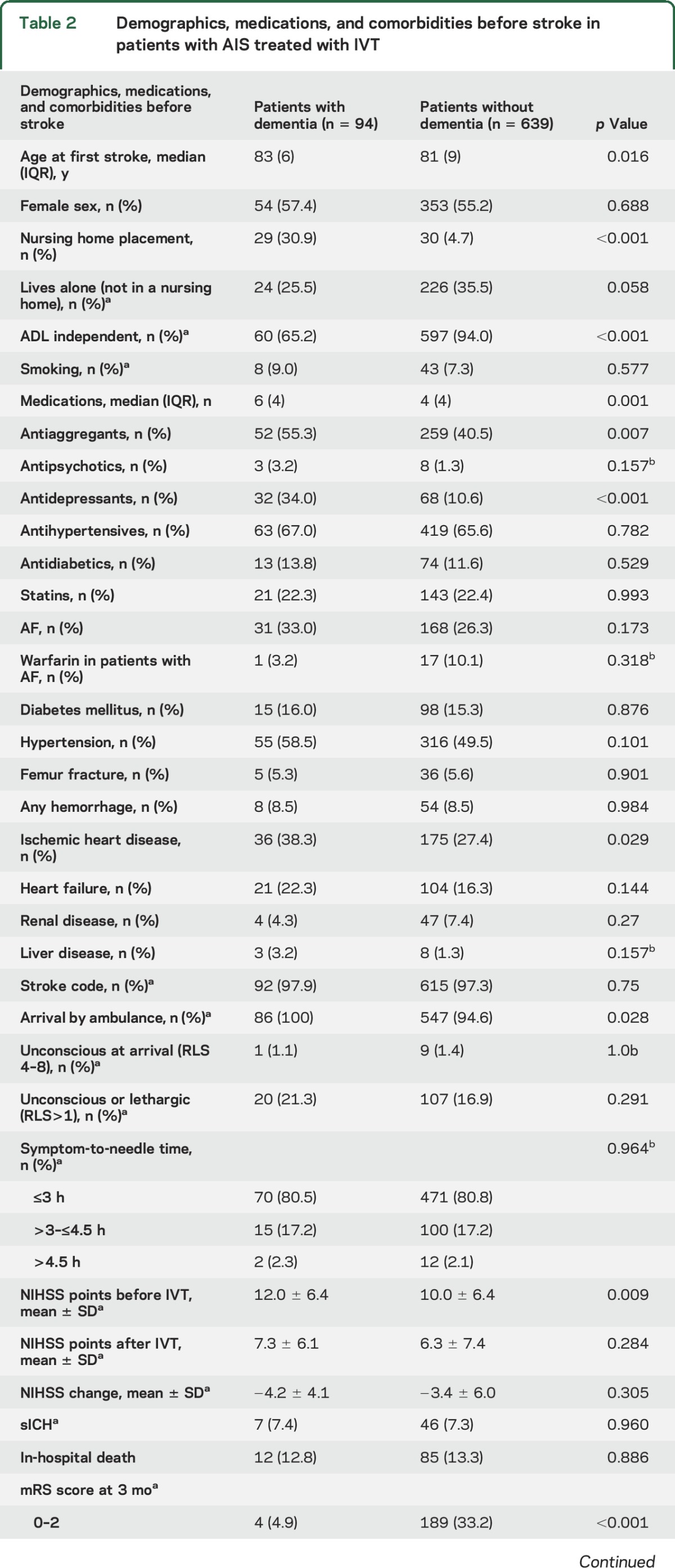

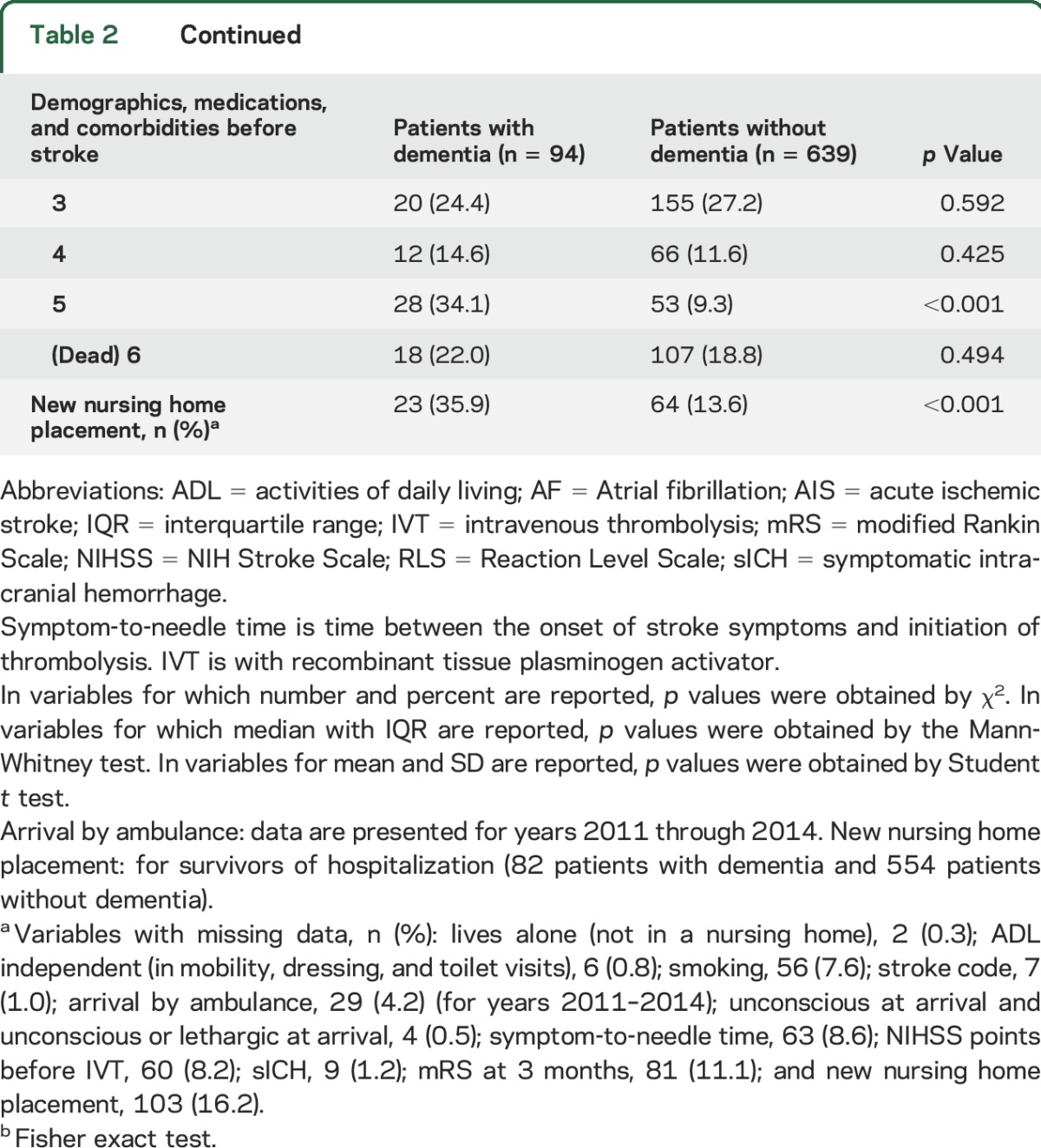
Demographics, medications, and comorbidities before stroke in patients with AIS treated with IVT

Symptom-to-needle time did not differ between patients with and those without dementia (table 2). Patients with dementia had a higher NIHSS score before IVT (12 vs 10 points, *p* = 0.009). This difference in NIHSS score was no longer present in the evaluation after IVT treatment (*p* = 0.284); however, the proportion of missing data was 29.3%. There were no differences in sICH, in-hospital death, and death at the 3-month follow-up (table 2), but 56.1% of patients with dementia (compared to 18.8% of controls) presented with an mRS score of 5 or 6 at 3 months. Thrombectomy was performed in 3 (3.2%) patients with dementia and 37 (5.8%) patients without dementia receiving IVT (*p* = 0.307) (results not presented).

ORs for patients with dementia receiving IVT are presented in table 3. In the fully adjusted covariate model (model 2) and in the post hoc propensity score–adjusted model (model 3), dementia was associated with lower odds of receiving thrombolysis. However, when the analysis was repeated exclusively among patients who were ADL independent before stroke, the difference between patients with and without dementia was no longer significant for the whole cohort (OR 0.79, 95% CI 0.60–1.06), although differences persisted for patients ≤80 years of age (table 3).

**Table 3:**
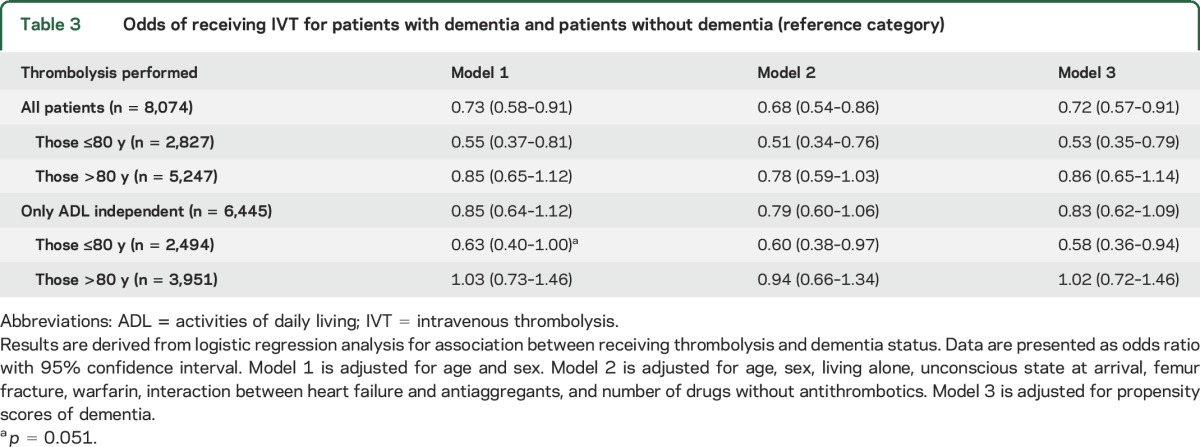
Odds of receiving IVT for patients with dementia and patients without dementia (reference category)

### Thrombolysis outcomes.

In the fully adjusted model, there were no differences in OR for death at the 3-month follow-up between patients with and those without dementia who received IVT. Functional outcome at 3 months, assessed with mRS, was worse among patients with dementia. In the fully adjusted covariate model, the OR for a higher mRS score in dementia was 3.65 (95% CI 2.06–6.45). In patients with dementia, OR for new nursing home placement was tripled (table 4).

**Table 4:**
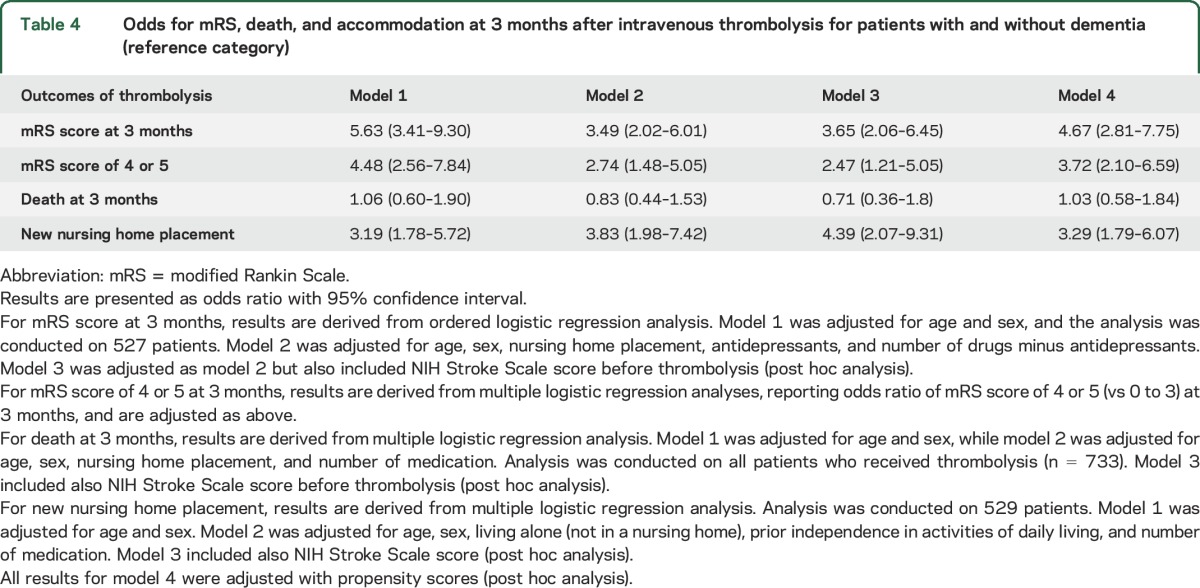
Odds for mRS, death, and accommodation at 3 months after intravenous thrombolysis for patients with and without dementia (reference category)

## DISCUSSION

In this large, nationwide, longitudinal study, we observed the following key findings: (1) patients with dementia were less likely to receive IVT, but these differences disappeared in analyses focusing on previously independent patients, persisting only in patients ≤80 years of age; (2) the frequencies of sICH and in-hospital and 3-month mortality after IVT were similar in patients with dementia and controls; and (3) among patients who received IVT, dementia was associated with greater disability and new nursing home placement.

Before stroke, patients with preexisting dementia had more comorbidities and were more ADL dependent, which is in line with previous studies from SveDem.^21^ The proportion of IVT-treated patients (9.5% for those without dementia and 7.0% for patients with dementia) was slightly higher than other recent national averages (6.4% in New Zealand in 2016,^22^ 6.1% in the United States^23^).

Patients treated with IVT (those with dementia and controls) were more independent than their counterparts who did not receive IVT. Moreover, ADL independence acted as a mediator for receiving IVT; hence, it probably represents an important decision factor for a physician contemplating IVT treatment, which is in line with the current guidelines.^3,19^

Worse functional prognosis after stroke in older patients^24^ and in those with dementia^7,8^ could lead to therapeutic nihilism and withholding of treatment. On the other hand, excessive enthusiasm for IVT could expose individuals to serious complications. Prior studies on IVT in AIS and dementia investigated death and sICH,^7–9^ and to the best of our knowledge, only one study specifically addressed functional outcomes after IVT.^10^ In that study, the proportion of independent patients with dementia in the initial cohort (28.9%) and in the cohort who received IVT (48.5%) was lower compared to ours (52.8% and 65.2%, respectively). This may be due to different definitions of independence or to the fact that dementia diagnosis in their study originated from clinical records (possibly including later dementia stages), whereas we included patients at the time of dementia diagnosis.^11^ In SveDem, >60% of patients are diagnosed with dementia with an MMSE score of ≥20.^12^ In our study, patients with dementia received the same standard of care regarding transport by ambulance and speedy initiation of IVT, but younger patients with dementia were less likely to have an activation of stroke code.

In line with previous studies,^25^ patients with dementia and AIS who received IVT were more selected, which is supported by the lower IVT rate in this group compared to controls. It seems that the decision criteria in both patients with and without dementia were functional status and age, because the difference could not be attributed solely to greater comorbidities and resultant contraindications, for which we adjusted. However, there might still be some residual confounding for which we could not account. Current American guidelines for IVT in dementia are subject to individual judgment, and they suggest taking into account life expectancy, premorbid functional level, and clinically meaningful benefit.^3^

Some studies found that mortality after stroke in dementia is increased regardless of the use of IVT,^7,8^ which could be partly explained by worse baseline status.^26^ A recent SveDem study showed that stroke was a substantial cause of death in dementia.^27^ The present study did not find differences in mortality in IVT-treated patients with dementia during hospitalization or at the 3-month follow-up, but functional outcomes were worse. The incidence of sICH was similar (7.4% vs 7.3%, *p* = 0.960).

We found no differences between the 2 groups in improvement in NIHSS score, but the proportion of missing data was high (≈30%). The initial difference in NIHSS could not be explained by the degree of consciousness, which was similar between groups, but might be due to greater stroke severity, confusional state, or poorer understanding of instructions in patients with both dementia and AIS.

IVT-treated patients with dementia had worse functioning at the 3-month follow-up. In a study from 2012,^10^ dementia itself was not an independent predictor of a worse functional outcome. However, the groups were matched for prior residence and preadmission dependency, whereas we adjusted only for residence. Besides dementia, factors associated with poor outcome in our model were age, prior nursing home placement, and total number of medication. Consistent with a previous study based in the United States,^7^ our IVT-treated patients with dementia had an unfavorable discharge destination compared to controls. In our study, dementia was independently associated with a new nursing home placement, a finding that differs from previous studies.^10^ The odds for patients with dementia of being placed in a long-term facility were increased (OR 3.83, 95% CI 1.98–7.42).

A multicenter European study investigated the effects of preexisting dependency (often dementia) on outcomes and complications of IVT.^26^ Dependent patients presented higher mortality rates; however, in our study, the incidence of poor outcome in survivors did not differ. Thus, previously dependent patients might still benefit from IVT.

This study has several limitations. First, baseline functioning in our cohort could be assessed only indirectly from degree of independence and living situation, while only an mRS estimation was available for outcome. Other methods of measuring functioning could be of interest, but the study could include only those assessed in Riksstroke. Second, the percentage of missing NIHSS after thrombolysis was ≈30%, limiting its usefulness. Third, nursing home residents with dementia and AIS are probably underrepresented in this study because they may be less often referred to hospitals. Causal inference is limited in cohort studies. IVT is an established treatment with a known risk-benefit profile. Our study shows that sICH and mortality outcomes were similar between patients with dementia and patients without dementia, although functional outcomes were worse. This may assuage some concerns when IVT is administered to this specific population. Those with dementia were eliminated from the control group by the exclusion of patients who were ever registered in SveDem, received a dementia or confusion diagnosis in the Swedish National Inpatient Register, or took antidementia drugs, but perfect case ascertainment is impossible. Riksstroke offers a >90% national coverage of ischemic stroke events, and the national dementia registry, SveDem, provides superior accuracy in dementia diagnoses compared to claims data. However, in 2012, the estimated coverage of incident dementia cases in SveDem was 36%,^11^ and the data on cognitive status (MMSE) were obtained at the time of dementia diagnosis, a median of 1.5 years before the stroke. SveDem may not be representative of the general dementia population, and dementia severity at the time of stroke is impossible to ascertain. SveDem diagnoses are not externally validated, but only ≈5% of patients change diagnoses at follow-up, suggesting that the initial diagnoses are robust.^12^ Patients may refuse participation in SveDem or Riksstroke, and no information is collected on nonincluded patients. However, inclusion is the default, and refusal is generally low in our clinical experience. The absence of dementia among the control group was ascertained through SveDem and other registries, but further exclusion of dementia cases (by examining patients or their journals) was not attempted and is an important limitation of this study.

To the best of our knowledge, there have been no studies on this topic since the prolongation of the IVT treatment window to 4.5 hours in 2009, and we are the first to report symptom-to-needle time in this group of patients. The large nationwide cohort, detailed dementia and stroke characterization, and low proportion of missing (including for the 3-month follow-up) are strengths of our study.

The present study, investigating use of IVT and its outcomes in dementia, suggests that in selected patients IVT is safe, with rates of treatment complications similar to those in patients without dementia. Patients with dementia have worse functional outcomes after IVT, which might be explained by worse baseline functional status.

## GLOSSARY

ADL: activities of daily living
AIS: acute ischemic stroke
ATC: Anatomical Therapeutic Chemical
CI: confidence interval
ICD-10: *International Classification of Diseases, 10th revision*
IVT: intravenous thrombolysis
MMSE: Mini-Mental State Examination
mRS: modified Rankin Scale
NIHSS: NIH Stroke Scale
OR: odds ratio
Riksstroke: Swedish national quality registry for acute vascular diseases of the brain
RLS: Reaction Level Scale
sICH: symptomatic intracranial hemorrhage
SveDem: Swedish Dementia Registry
